# Health Resort Treatment Mitigates Neuropsychiatric Symptoms in Long COVID Patients: A Retrospective Study

**DOI:** 10.3390/healthcare13020196

**Published:** 2025-01-19

**Authors:** Grzegorz Onik, Katarzyna Knapik, Dariusz Górka, Karolina Sieroń

**Affiliations:** 1 Department of Physical Medicine, School of Health Sciences in Katowice, Medical University of Silesia in Katowice, 40-752 Katowice, Poland; kknapik@sum.edu.pl (K.K.); ksieron@sum.edu.pl (K.S.); 2 Department of Sports Medicine and Physiology of Physical Effort, School of Health Sciences in Katowice, Medical University of Silesia in Katowice, 40-752 Katowice, Poland; dgorka@sum.edu.pl

**Keywords:** long COVID, health resort treatment, persistent symptoms, rehabilitation, neurological disorders, psychiatric symptoms, spa treatment, complementary and alternative medicine

## Abstract

Background/Objectives: Among the neuropsychiatric symptoms of long COVID, the following may be listed: sleep disturbances, headaches, anxiety, depression, dizziness, numbness, memory loss, and concentration difficulties. Various therapies have been implemented to mitigate these symptoms; however, health resort treatments that utilize a wide range of modalities stimulating multidirectional biological reactions may also be effective. The aim of this study was to assess the severity of neuropsychiatric symptoms in long COVID patients who qualified for health resort treatment, evaluate the effectiveness of health resort treatment in this group of patients, and evaluate the effect of balneological factors in the treatment course. Methods: A retrospective analysis of the medical records of 120 people with long COVID (69 women and 51 men) aged 42–79 who underwent health resort treatment in 2021 was performed. People were eligible for treatment at a lowland health resort based on a valid referral from a doctor. The treatment included balneological therapies, physical medicine modalities, exercise programs, health education, and psychological support. Patients assessed the severity of persistent neuropsychiatric symptoms on a 0–10 point scale before and after treatment. Results: After the treatment, the greatest improvement was noted in sleep disorders (2.47 ± 2.23 points vs. 0.86 ± 1.25 points, *p* < 0.00001) and dizziness (1.39 ± 1.94 points vs. 0.34 ± 0.76 points, *p* < 0.00001). The lowest improvement was observed in memory disorders (2.68 ± 2.5 points vs. 1 ± 1.4 points, *p* < 0.00001). Conclusions: Patients with long COVID who qualified for health resort treatment reported mild neuropsychiatric symptoms. Health resort treatment mitigates neuropsychiatric symptoms, as it is a complex approach. Treatment that includes balneological factors improves symptoms to a greater extent. This method of treatment should be integrated into the standard treatment for long COVID.

## 1. Introduction

The World Health Organization defines long COVID as a condition occurring in patients with a history of probable or confirmed SARS-CoV-2 infection that typically develops 3 months after the onset of symptoms, persists for at least 2 months, and cannot be explained by alternative diagnoses [1]. In long COVID patients, various symptoms may persist, including those affecting the vascular system, respiratory system, musculoskeletal system, gastrointestinal tract, urinary system, and nervous system [1,2]. Neurological symptoms arise from damage to the central and peripheral nervous systems, which is why diverse neurological symptoms may be observed in COVID-19 survivors. Among these, the most frequently reported are fatigue, sleep disturbances, headaches, anxiety, depression, dizziness, numbness, ageusia, altered taste, postural instability, memory loss, concentration difficulties, and vision impairment. The prevalence of symptoms varies depending on the report. Nevertheless, in long COVID patients, fatigue, sleep disorders, and headaches are the most commonly observed symptoms [1,2,3,4,5,6].

The exact pathomechanism of persistent neurological symptoms in long COVID patients is still debated. It is postulated that coagulopathy, resulting from inflammation, cellular damage, and vascular dysfunction, leads to insufficient tissue oxygenation and may thus contribute to long-lasting symptoms in COVID-19 survivors [5,7,8]. Other factors that might impact prolonged neurological symptoms include thrombosis and disruption of the blood–brain barrier [5,9]. Moreover, chronic inflammation is also linked to long-term symptoms [5,7,8]. Grisanti et al. [9] suggest that neuropathies are inflammation-dependent, as inflammation may be a manifestation of nerve damage caused by the SARS-CoV-2 virus or an autoimmune reaction. Angiotensin-converting enzyme 2 receptors also play a significant role in the neurological sequelae of COVID-19. Since these receptors are distributed in different parts of the central nervous system, particularly in the brain stem, the presence of certain symptoms may be associated with the involvement of specific sites rich in these receptors [3]. Additionally, since neurons have limited regenerative ability, this may also contribute to the long-term neurological symptoms in individuals who have recovered from COVID-19 [2]. Furthermore, some reports also indicate disturbances in the brain glymphatic system] [10] and autonomic dysfunction [11] as factors contributing to persistent neurological symptoms in long COVID patients. Considering the various mechanisms determining the neurological sequelae of COVID-19, patients may exhibit various symptoms; therefore, a complex approach to alleviate them is required.

Long COVID is a reason for the public health and economic burden, particularly among elderly people [12]. Mirin [13] estimated that in the USA, the annual medical costs associated with long COVID range from USD 43 billion to USD 172 billion. Therefore, different treatment strategies have been implemented to reduce the severity of persistent COVID-19 symptoms, including rehabilitation [2,10], exercise [] [14,15], dietary supplements [7,16], pharmacotherapy [2,17,18,19], aromatherapy [20], brain stimulation [21], and hyperbaric oxygen therapy [22]. Thurner et al. [23] suggest that neurocognitive training and individual psychotherapy should also be implemented, as patients with long COVID experience not only physical symptoms. In Poland, the costs of post-COVID rehabilitation were funded by public resources, in accordance with a decree of the president of the National Health Fund (63/2021/DSOZ). Patients were qualified for the post-COVID rehabilitation program based on a valid referral provided by a health insurance doctor.

It is suggested that balneotherapy may effectively mitigate long COVID symptoms [24]. Health resort treatment is a complex therapy, not only due to the application of balneological factors (mineral waters, mud therapy, gas therapies) and climatotherapy but also because of the use of various procedures, including hydrotherapy, massage, exercise, and physical medicine modalities. Other key elements of this treatment approach are health education, psychotherapy, and diet [24,25,26,27,28,29]. Health resort treatment can be carried out in various environments, such as seaside, mountain, upland, and lowland areas [30,31], where the natural surroundings promote healing. Regardless of the sanatorium’s location, recreation, cultural, and social aspects also influence patients during the treatment course [29]. Treatment applied in sanatoria has been shown to reduce inflammation, ease pain, modulate the immune system, modify hormone activity, improve blood flow and hemorheology, alter oxidative status, and influence the autonomic nervous system [32,33,34,35,36]. Considering hypotheses explaining persistent COVID-19 symptoms, complex health resort treatment may be effective [5,9,10,11]. Health resort treatment has few side effects when the approach is personalized [37]. Compared to pharmacotherapy for long COVID, it may be more favorable due to its multidirectional action, as the heterogeneity of post-COVID syndrome tends to be a limitation of pharmacotherapy [2].

Until now, few studies have evaluated the effects of health resort treatment in post-COVID patients [38,39,40,41]. However, Costantino et al. [41] have proven that spa therapy reduced anxiety, depression, and stress, as well as improved sleep quality in patients with a history of COVID-19. García-López et al. [33] reported that balneotherapy improves depression, while Masiero et al. [26] postulated that it may also mitigate anxiety and mental stress. Nevertheless, no study has assessed the effects of health resort treatment on the severity of concentration and memory disorders, dizziness, and paresthesia. This is why our study may be informative and help fill a gap in knowledge. Considering the favorable effect of balneotherapy, we also decided to verify its superiority over non-balneological approaches in mitigating long COVID neuropsychiatric symptoms during treatment in a sanatorium.

This study had three aims. The first was to assess the severity of concentration disorders, memory disorders, headaches, dizziness, sleep disorders, paresthesia, depression, and anxiety in long COVID-19 patients who qualified for health resort treatment. The second was to assess the effectiveness of the complex treatment applied in the health resort to this group of patients. The third was to compare the efficacy of treatment based on the application of balneological factors.

## 2. Materials and Methods

### 2.1. Eligibility Criteria

The inclusion criterion was a complete medical record, including demographic data, medical history, description of the treatment course, and levels of concentration and memory disorders, headaches, dizziness, sleep disorders, paresthesia, depression, and anxiety.

The following exclusion criteria were set: age above 80 years, neuropsychiatric disorders (multiple sclerosis, post-stroke syndrome, Parkinson’s disease, epilepsy, depression), cardiovascular diseases (coronary artery disease, heart failure, myocardial infarction history, percutaneous coronary interventions and/or coronary artery bypass grafting history, endarterectomy history, pacemaker implantation, atrioventricular and/or bundle of His blocks, atrial fibrillation, peripheral artery disease), respiratory system disorders (chronic obstructive pulmonary disease, emphysema, pneumoconiosis, asthma), rheumatic diseases (rheumatoid arthritis, ankylosing spondylitis), cancer, lower limb amputations, blindness, Lyme disease, and inability to undertake general development exercises. These conditions were considered confounders that could impact the study results and diminish the study’s reliability.

### 2.2. Data Collection

We reviewed the data on 239 patients undergoing health resort treatment for long COVID at the Gwarek Rehabilitation Hospital and Sanatorium in Goczałkowice-Zdrój (Poland) in 2021. The town is situated in the Oświęcim Valley, making it a lowland resort with an area of 4864 ha. Health resort status was awarded to the town in 2016 [42,43]. Evaluation of long COVID symptom severity before and after treatment was required by the Polish post-COVID rehabilitation program. The data were not originally collected for scientific purposes. After obtaining the positive opinion of the authorities of the Gwarek Rehabilitation Hospital and Sanatorium, patient data were anonymized and then reviewed. Therefore, no informed consent was required. Medical records were reviewed between March and May 2024. The Bioethical Committee of the Medical University of Silesia in Katowice stated that this project did not require its opinion (decision BNW/NWN/0052/KB/238/23).

### 2.3. Subjects

**Figure 1:**
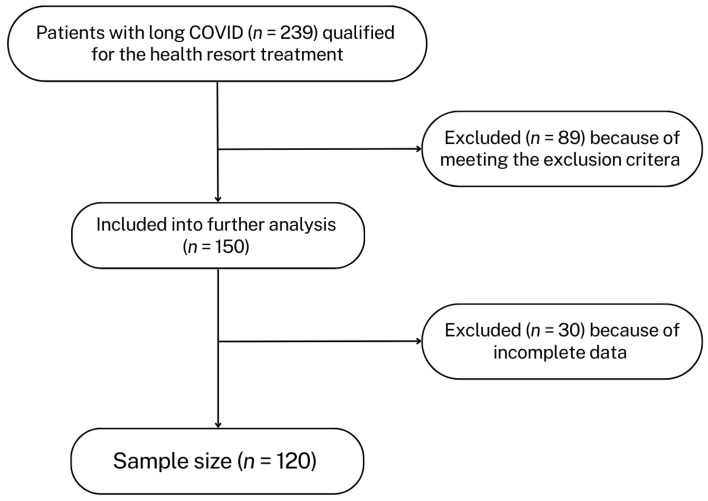
Schematic representation of data selection for analysis.

After considering the exclusion criteria, 150 medical histories were included in the next stage of review. Due to incomplete data, 30 medical records of patients undergoing health resort treatment were excluded. In the final stage, medical records of 120 patients were analyzed (Figure 1).

### 2.4. Treatment Course

The treatment course was individually tailored based on the analysis of clinical symptoms during the examination upon admission. The treatment was not standardized, and varied among patients depending on their condition and needs. Nevertheless, according to the Polish post-COVID rehabilitation program, each patient was required to attend at least four procedures each day during treatment. In total, the treatment course involved performing 96 procedures during the stay at the sanatorium. The treatment was complex due to the application of various methods, including balneotherapy, general developmental and respiratory exercises, physical medicine modalities, and health education. Respiratory exercises aimed to relax chest muscles, improve diaphragmatic breathing, activate the lower ribs, and facilitate prolonged exhalation. Meanwhile, general developmental exercises were designed to train dynamics, endurance, balance, coordination, and muscle strength. A physiotherapist supervised all exercises. Each patient underwent a routine control examination after seven days of treatment to verify a favorable reaction to the applied treatment and to exclude side effects. All patients attended health education sessions aimed at promoting a healthy lifestyle and supporting recovery from addiction, if applicable. Moreover, all patients also received psychological support. After completing the treatment, patients were discharged. The treatment duration varied, as the rules of the Polish post-COVID rehabilitation program stipulated an intervention of 2 to 6 weeks.

### 2.5. Symptom Severity Evaluation

Prior to the long COVID symptom severity assessment, patients were instructed on how to use the numeric rating scale. Participants were informed that they should assess the severity of symptoms at the time of evaluation. Additionally, patients were instructed to state the number that most reliably represented the severity of particular symptoms, with the assumption that 0 indicated “no symptoms” and 10 represented “the most severe symptoms imaginable.” We adopted the following method of interpretation: 0—no symptoms; 1–3 points for mild symptoms, 4–7 points for moderate symptoms, and 8–10 points for severe symptoms.

### 2.6. Statistical Analysis

Statistical analysis was performed with STATISICA 13 PL software. The effect size was calculated using G Power 3.1.94 software. With the assumption of a sample size of 120, α = 0.05, and statistical power (1-β error probability) = 0.95, the effect size was established as ƒ = 0.309. Data normalcy distribution was checked with the Shapiro–Wilk test. Intragroup comparisons were performed using the Wilcoxon signed-rank test. Intergroup comparisons (between genders and groups of patients depending on application of balneological factors) were carried out with the Mann–Whitney U test. Moreover, we calculated delta values (∆), expressed as the difference between pre- and post-treatment assessments, to establish the magnitude of symptom changes. The statistical significance level was set at *p* < 0.05.

## 3. Results

Medical records of 120 people with long COVID (69 women and 51 men) aged 42–79 years (mean age: 64.21 years ± 8.67 years) were analyzed. The patients’ mean body mass was 85.91 kg ± 15.27 kg, mean body height was 1.67 m ± 0.09 m, and mean body mass index was 30.71 kg/m^2^ ± 5.09 kg/m^2^. In the reviewed individuals, mean systolic blood pressure was 139.63 mmHg ± 13.34 mmHg, and diastolic blood pressure was 80.08 mmHg ± 7.55 mmHg, measured upon admission. Treatment duration ranged from 17 to 47 days (mean treatment duration: 24.44 days ± 6.24 days). Patients’ characteristics are presented in Table 1.

In patients who qualified for the health resort treatment, hypertension was the most common coexisting disease (58.33%, *n* = 70). In hypertensive patients, mean systolic blood pressure was 141.8 mmHg ± 13.12 mmHg and diastolic blood pressure was 80.27 mmHg ± 8.16 mmHg upon admission. Type 2 diabetes mellitus was coexisting in 18.33% of the individuals (*n* = 22). In diabetic patients, mean glycemia was 120.42 mg/dl ± 17.27 mg/dL. Degenerative joint disease was noted in 21.67% of patients (*n* = 26). Hypothyroidism was diagnosed in 12.5% of patients (*n* = 15). Gout was observed less frequently (3.33% of patients, *n* = 4). Only one man was diagnosed with benign prostatic hyperplasia.

In patients undergoing health resort treatment for long COVID, the most frequently applied modality was pneumatic massage (65.83%, *n* = 79). Among physical medicine modalities, low-level laser therapy (60%, *n* = 72) was the most frequently administered. Water therapies were applied less frequently. Mud therapy was prescribed to 13.33% of patients (*n* = 16). Only six individuals (5%) underwent ultrasound therapy (Table 2).

Most long COVID patients reported that persistent neuropsychiatric COVID-19 symptoms were of mild intensity. Only 4% of individuals were affected by severe concentration and memory disorders, as well as paresthesia (Table 3).

During the pre-treatment assessment, women reported greater intensity of persistent neuropsychiatric COVID-19 symptoms. Upon admission, women had approximately 16% more concentration problems and about 39% more memory disorders. Depression severity was about 37% higher in women who qualified for health resort treatment compared to men. Additionally, women experienced more headaches and dizziness, with an increase of about 50% and 58%, respectively, compared to men. After treatment at the health resort, the severity of symptoms did not differ between genders. However, the health resort treatment led to a significant reduction in all persistent COVID-19 symptoms. In the entire group of patients, the highest decrease in symptom severity was noted for dizziness (approximately 75% reduction), while the lowest decrease was observed for memory disorders (approximately 63% reduction). After treatment, women reported the greatest reduction in dizziness (approximately 79%), paresthesia (approximately 73%), and depression (approximately 71%). Memory disorders showed the lowest improvement in women (approximately 64%). In men, the treatment led to significant improvements in anxiety (approximately 82% reduction), paresthesia (approximately 77% reduction), and headache (approximately 77% reduction). The lowest effectiveness of the treatment was observed in sleep disorders (approximately 60% improvement) (Table 4). A comparison of the magnitude of symptom changes revealed that in women, ∆memory disorders and ∆dizziness were approximately 44% and 65% greater than in men (Table 5).

The study group was divided into two subgroups based on the use of balneological factors during treatment at the sanatorium. Group I (*n* = 70) consisted of individuals treated with balneological factors (mud therapy, water therapies). Group II (*n* = 50) comprised individuals who were not exposed to natural healing resources. The only difference between the groups was diastolic blood pressure at admission; it was 3% higher in group II. The characteristics of patients included in groups I and II are presented in Table 6.

Before treatment, in group I, the severity of concentration disorders, paresthesia, and depression was significantly higher than in group II. At baseline, paresthesia was approximately 43% more severe in group I than in group II, while concentration disorders were approximately 38% more severe, and depression was about 27% more severe. After treatment, the severity of symptoms did not differ between the groups. In patients who received balneological treatments, the severity of all symptoms improved. The greatest reductions were noted in dizziness (approximately 77%), paresthesia (approximately 74%), and anxiety (approximately 72%). In group II, a similar pattern of symptom severity reductions was observed. The greatest improvement was observed in paresthesia (approximately 74%), headache (approximately 74%), and dizziness (approximately 73%) (Table 7).

In patients assigned to Group I, the magnitude of symptom changes was greater than in those not treated with balneological factors. The change in concentration disorders (∆concentration disorders) was approximately 41% greater in Group I than in Group II. The change in memory disorders (∆memory disorders) was approximately 36% greater in Group I compared to patients in Group II. The largest difference between the groups was observed in ∆paresthesia (approximately 44% difference). The smallest difference between the groups was noted in ∆anxiety (approximately 34%) (Table 8).

## 4. Discussion

### 4.1. Symptom Occurrence

In patients who qualified for health resort treatment, the incidence and severity of neuropsychiatric symptoms varied. The most frequently reported symptom was paresthesia, with 74.2% of patients experiencing numbness or tingling in the upper or lower extremities. Our results are inconsistent with the data presented by Pilotto et al. [44], who reported that the prevalence of numbness/tingling in patients six months after recovery from COVID-19 was 18.8%. Among patients undergoing health resort treatment for long COVID, 48.37% described the severity of paresthesia as mild. It is hypothesized that pro-inflammatory cytokines released during the acute phase of SARS-CoV-2 infection may cause hypersensitization of peripheral nociceptors [45]. This pathomechanism could explain the persistent paresthesia observed in the analyzed patients. During the pre-treatment examination, 70% of individuals reported sleep disorders. These results contrast with those of Pilotto et al. [44], who reported that 31.5% of post-COVID patients experience sleep disturbances. Additionally, Pinzon et al. [46] provided similar data, indicating that sleep disorders as a neurological sequela of COVID-19 affect 32% of individuals. Nevertheless, among patients undergoing health resort treatment, the majority reported mild severity (38.3%). In long COVID, sleep disturbances may result from several mechanisms; however, the most probable cause appears to be disruption of the circadian rhythm, which is a consequence of damage to neurons in the sleep-regulating areas of the brain [47].

Overall, 68.3% of patients reported memory disorders upon admission to the sanatorium. Our data are comparable to those presented by Davis et al. [48], who reported that short- and long-term memory problems occurred in 72.81% of COVID survivors, with prevalence decreasing over time. Similar data were presented by Stefanou et al. [3], who reported that memory impairment affected 73% of post-COVID patients. Among patients undergoing health resort treatment, 35% reported moderate memory disturbances. Probable mechanisms for memory impairment in post-COVID patients include a reduction in gray matter, infection-mediated hippocampal damage, and brain tissue hypoxia [49]. In our cohort, 62.5% of patients reported concentration disorders upon admission. In patients qualified for health resort treatment, the incidence of concentration problems was lower than that reported by De Luca et al., who found that 77.8% of patients experienced them. The underlying mechanism of cognitive dysfunction in long COVID patients is believed to involve inflammation-mediated neuronal damage and neurodegeneration [50]. Similarly to memory impairment, concentration disorders were assessed as moderate in most patients. In sum, 50.83% of patients reported depression during the pre-treatment examination, though no patient experienced severe depression symptoms, while 46.67% of long COVID patients reported anxiety, with most of them reporting mild severity. Stefanou et al. [3] reported that depression and anxiety affected 27% of COVID-19 survivors six months after infection clearance. In our study, the prevalence of these symptoms was higher. However, most patients reported mild depression and anxiety, at 35.83% and 37.47%, respectively.

Overall, 42.5% of patients reported headaches upon admission, with most of them assessing the severity as mild (25.5%). Our results contrast with data provided by Davis et al. [48], who reported that headaches occurred in 77–78% of patients with long COVID. In contrast, Pilotto et al. [44] reported that, six months after recovery from COVID-19, headaches were reported by 9.7% of patients. Moreover, Caronna et al. [51] found that headaches in long COVID patients occur most frequently in those above 45 years of age. In our study, the mean age of participants was 63.45 ± 8.78 years, which supports our finding regarding the occurrence of headaches in long COVID patients. However, we did not observe a correlation between age and headache severity (r = 0.01; *p* < 0.05). The frequency of dizziness was comparable to that of headaches, affecting 41.7% of individuals. In our study, we observed a higher incidence of dizziness compared to Lopez-Leon et al. [52], who reported that dizziness affected 3% of long COVID patients. It is postulated that headaches and dizziness result from microemboli in the brain, blood–brain barrier insults, and inflammation of neural tissue [5].

The comparison of pre-treatment symptom severity between women and men revealed that females had significantly greater intensity of most neuropsychiatric symptoms. This is consistent with reports suggesting that women are more prone to developing long COVID symptoms [1,8,12]. Zakia and Pradana [53] suggested that women, especially those who are obese, are at higher risk of sleep disturbances. In our study, the mean body mass index in women was 31.46 ± 5.9 kg/m^2^; however, we did not observe a correlation between this variable and the severity of sleep disorders upon admission (r = 0.01; *p* < 0.05). Moreover, the severity of sleep disturbances did not differ significantly between women and men during the pre-treatment assessment (*p* = 0.15). The greatest difference between genders during admission was noted in dizziness and headaches. The severity of dizziness and headaches was greater in women upon admission. Rodrigues et al. [54] reported that headaches occur more frequently in women and are more intense, which is consistent with our results. The severity of concentration disorders and memory impairment was greater in women. Fernández-de-Las-Peñas et al. [55] did not find an association between female sex and memory disturbances or concentration impairment. This contradicts our results; however, it might be a consequence of using different measurement tools. In individuals undergoing health resort treatment due to long COVID, women reported more severe depression compared to men. This finding is consistent with Rudenstine et al. [56], who demonstrated that women reported higher odds of probable depression outcomes.

### 4.2. Treatment-Mediated Symptom Alleviation

The complex treatment applied during care in the sanatorium reduced the severity of persistent COVID-19 symptoms in all patients. Latorre-Román et al. [57] demonstrated that balneotherapy improved sleep quality and reduced depression, which aligns with our findings. Moreover, balneotherapy effectively reduces stress levels. Treatment-mediated relaxation may be a consequence of changes in the endocrine system, especially cortisol levels and endogenous opiates [41,58]. Additionally, the alleviation of depression and anxiety may also be associated with the experience of staying in a beautiful landscape surrounded by greenery and the absence of work-related stressors [59,60]. Costantino et al. [41] postulate that social relationships may also improve the health status of post-COVID patients and as a consequence reduce depression and anxiety. Furthermore, Castelli et al. [61] claim that balneotherapy may influence thermoregulation, which plays a role in the sleep–wake cycle, thus explaining the improvement in sleep quality in patients undergoing health resort treatment. This could explain the improvement observed in individuals who underwent health resort treatment for long COVID.

Considering the inflammation-dependent nature of neuropathies [9], the application of balneological factors may be effective, as they reduce the level of pro-inflammatory cytokines while increasing the levels of anti-inflammatory cytokines [62]. The anti-inflammatory effect may also address headaches in long COVID patients, as pathomechanism assumes persistent immune system activation [19]. Moreover, after COVID-19, memory disorders may result from inflammation in the medial temporal lobe and neural tissue atrophy [63]. Poor neuronal regeneration may be supported by the regenerative action of balneological factors, which have been proven to stimulate tissue repair and angiogenesis [64]. This may explain the reduction in paresthesia and memory disorders in patients undergoing health resort treatment.

In patients with long COVID, autonomic dysregulation may be observed. Treatment with balneological factors may influence the autonomic nervous system and mediate health status, contributing to improvements in individuals attending a sanatorium for long COVID treatment [65,66]. During their stay in the sanatorium, patients regularly participated in exercises, including activities aimed at improving balance. This may have contributed to the reduction in dizziness, as rehabilitation has already been proven to be an effective intervention [63].

To summarize, health resort treatment can significantly improve the health of long COVID patients, not only those experiencing neuropsychiatric symptoms but also those with cardiopulmonary impairments and functional deficits [38,39,40]. As a complex intervention, health resort treatment triggers the regeneration of neural tissue, reduces inflammation, stimulates the immune system, improves blood circulation, and modifies the hormone system, which may effectively address neuropsychiatric symptoms in long COVID [32,33,34,35,36,57,58,59,60,61,62]. Moreover, Kardeş [37] postulates that health resort treatment may contribute to diminishing the economic burden of COVID-19 on the healthcare system, especially in Europe, due to the high accessibility of this treatment as well as its acceptance among patients.

### 4.3. Gender-Dependent Treatment Outcomes

To establish sex-dependent differences in treatment outcomes, we calculated the deltas. We observed that in women, the magnitude of changes in memory disorders and dizziness was greater than in men. Kurre et al. [67] suggest that disability, anxiety, and depression associated with dizziness are gender-dependent, and therefore treatment strategies should differ. The treatment course in the sanatorium was individually tailored rather than gender-tailored, so it cannot explain the observed differences between women and men. Yang et al. [68] reported that the length and frequency of treatment were factors influencing outcomes in women undergoing hot spring balneotherapy. Therefore, we examined the influence of treatment duration on delta values. In women undergoing health resort treatment for long COVID, we found significant correlations between treatment duration and ∆memory disorders (r = 0.27; *p* < 0.05) and ∆dizziness (r = 0.38; *p* < 0.05). Similarly, in men, significant correlations between treatment duration and ∆memory disorders (r = 0.42; *p* < 0.05) and ∆dizziness (r = 0.36; *p* < 0.05) were observed. As we noted significant correlations in both genders, treatment duration cannot explain the differences in delta values between women and men. Fankhauser [69] reports that hormonal and neurotransmitter fluctuations play a role in the pathomechanism of neuropsychiatric disorders in women. Moreover, Antonelli et al. [59] reported that balneotherapy may improve stress resilience by influencing cortisol levels. This may explain the obtained results, as this form of treatment affects hormone levels [62,70].

### 4.4. Balneology-Dependent Treatment Outcomes

We decided to divide individuals into two groups based on the use of balneological factors (water therapies, mud therapy) to mitigate long COVID symptoms. Significant improvement in neuropsychiatric symptoms was observed in both patients treated with balneological factors and those not exposed to natural healing factors. Nevertheless, a comparison of deltas revealed that in patients treated with balneological factors, the magnitude of changes was greater for most symptoms, including concentration disorders, memory disorders, dizziness, paresthesia, and depression. Mineral waters and mud therapy have been shown to stimulate various effects, including vascular, analgesic, and anti-inflammatory effects [25,62,71]. Considering the pathomechanism of persistent COVID-19 symptoms, the application of natural healing factors may explain the obtained results [5,9,10,11].

The pre-treatment group comparison revealed no differences in age or anthropometric measurements, suggesting that these factors may not have influenced the results. Patients included in group I reported more severe long COVID symptoms at admission. This might be due to the group composition, as 61.43% of participants were women, who are more likely to suffer from long COVID [1,8,12]. Gordon et al. [72] reported that hypothalamic–pituitary–adrenal dysregulation may play a role in depression. Balneological factors apply heat, which may activate the hypothalamic–pituitary–adrenal axis, leading to a secondary increase in cortisol levels, mediating the reduction of inflammatory cytokines [62]. In fact, the anti-inflammatory effect of balneotherapy is a result of reduced concentrations of interleukin 1, prostaglandin E2, and leukotriene B4 [61]. As in long COVID patients, increased levels of interleukin 1β, interleukin 6, and TNF-α have been reported, and the mechanism explained above may account for the greater improvement in people exposed to natural healing factors. Moreover, activation of the hypothalamic–pituitary–adrenal axis and the subsequent release of β-endorphins may explain the analgesic effect observed in patients [62]. However, the change in headaches (∆headaches) did not differ between the groups. Furthermore, heat applied during mud therapy or water therapies stimulates vasodilation resulting from smooth muscle tension relaxation, which may also contribute to the analgesic effect [2,73,74,75].

There are few reports addressing the severity of balneology-mediated neuropsychiatric symptoms. Sujan et al. [76] have proven that natural healing factors reduce the intensity and frequency of headaches in migraine patients. The authors suggest that the analgesic effect is a consequence of the gate control theory, which assumes the inhibition of nociception through large-diameter myelinated fibers. It can be hypothesized that in long COVID patients undergoing balneotherapy, this mechanism may also contribute to the reduction in headaches. In post-stroke patients, Erceg-Rukavina et al. [77] demonstrated that balneological factors reduce spasticity on the affected side. One meta-analysis confirmed that hydrotherapy and balneology reduce depression and anxiety scores in adults; however, the paper rarely considered neuropsychiatric conditions [78]. In general, balneological factors are not widely applied in neuropsychiatric conditions, which is why our paper is informative, as it has shown that health resort treatment is an effective method. Our results suggest that in long COVID patients, standard rehabilitative procedures should be implemented alongside balneological factors to improve treatment effectiveness.

### 4.5. Potent Synergy with Standard Rehabilitation

Chuang et al. [10] suggest that long COVID requires a multidisciplinary approach and teamwork involving various medical professionals. Their proposal arises from the heterogeneity of long COVID and the diversity of its symptoms [1,2]. Nevertheless, it is recommended to implement health resort treatment alongside a pre- or post-admission rehabilitation program to enhance the effectiveness of balneotherapy in long COVID patients. This program should be exercise-based, as different types of exercises have been shown to mitigate persistent COVID-19 symptoms, improve functional capacity, and increase muscle strength with minimal adverse effects [2,10,79]. Balneological factors stimulate numerous biological effects [32,33,34,35,36,57,58,59,60,61,62], which may be enhanced by the effects of exercise in people with persistent COVID-19 symptoms.

Nopp et al. [80] developed a long COVID rehabilitation program and emphasized the importance of patient education, psychological support, diet modification, and smoking cessation. Health resort treatment for long COVID is a complex approach, as it includes all of these components. Thus, it should be viewed as a continuation of the treatment program within the patients’ living environment. In summary, health resort treatment is complementary to rehabilitation programs, incorporating all components routinely applied in rehabilitation courses. Combining standard rehabilitative procedures with health resort treatment in long COVID patients would lead to a synergistic effect, improve treatment efficacy, and reduce the economic burden on the healthcare system.

### 4.6. Study Limitations

Our study has several limitations. Firstly, we were unable to determine the exact period of time between SARS-CoV-2 infection clearance and the initiation of health resort treatment, as the Polish post-COVID rehabilitation program required treatment to begin within 12 months of recovery. Secondly, we used a numeric rating scale to evaluate the severity of symptoms, which may have low reliability when assessing neuropsychiatric symptoms. Future studies should use validated tools to assess specific symptoms. Thirdly, each patient received an individually tailored treatment course based on the pre-treatment examination. In future studies designed as randomized controlled trials, it would be appropriate to compare the favorable effects of balneotherapy-based treatment in long COVID patients with those receiving only exercise combined with physical medicine modalities. Moreover, these studies should follow a standardized treatment protocol. Fourthly, it would be valuable to assess the long-term effects of health resort treatment, with follow-up after 3 months.

## 5. Conclusions

Patients with long COVID who qualified for health resort treatment report mild neuropsychiatric symptoms. Balneological factors, exercises, and physical medicine modalities combined with mental health support and education mitigate neuropsychiatric symptoms in long COVID patients, with the greatest effectiveness in sleep disorders and dizziness. The implementation of balneological factors in the treatment course improves treatment effectiveness. Health resort treatment should be widely recommended as a complementary approach to the standard treatment of COVID-19 sequelae.

## Figures and Tables

**Table 1:** Characteristics of patients who qualified for the health resort treatment because of long COVID.

	Women (*n* = 69)			Men (*n* = 51)		
	Min	Max	Mean ± SD	Min	Max	Mean ± SD
Age (years)	43	77	64.77 ± 8.6	42	79	63.45 ± 8.78
Body weight (kg)	55	125	82.39 ± 16.32	70	120	90.67 ± 12.36
Body height (m)	1.5	1.78	1.62 ± 0.05	1.61	1.95	1.75 ± 0.07
BMI (kg/m^2^)	21.45	46.66	31.46 ± 5.9	23.99	39.79	29.7 ± 3.51
Systolic blood pressure (mmHg)	105	160	138.96 ± 14.49	115	165	140.53 ± 11.7
Diastolic blood pressure (mmHg)	55	95	78.96 ± 8.1	66	95	81.59 ± 6.49
Treatment duration (day)	19	47	24.97 ± 6.58	17	46	23.73 ± 5.74

**Table 2:** Procedures administered to patients undergoing health resort treatment because of long COVID.

Modality	*n*	%
Pneumatic massage	79	65.83
Low-level laser therapy	72	60
Cryotherapy	61	50.83
Infrared light therapy	49	40.83
Whirlpool baths	48	40
Electromagnetic fields	45	37.5
Electrotherapy	40	33.33
Pearl baths	27	22.5
Mud therapy	16	13.33
Classical massage	7	5.83
Ultrasound therapy	6	5
Lymphatic drainage	1	0.83

**Table 3:** Severity of neuropsychiatric symptoms in long COVID patients on admission.

Symptom	None *n* (%)	Mild *n* (%)	Moderate *n* (%)	Severe *n* (%)
Concentration disorders	45 (37.5%)	33 (27.5%)	38 (31.7%)	4 (3.3%)
Memory disorders	38 (31.7%)	26 (30%)	42 (35%)	4 (3.3%)
Headaches	69 (57.5%)	24 (35.5%)	26 (21.7%)	1 (0.8%)
Dizziness	70 (58.3%)	28 (23.4%)	22 (18.3%)	0 (0%)
Sleep disorders	36 (30%)	46 (38.3%)	35 (29.2%)	3 (2.5%)
Paresthesia	31 (25.8%)	58 (48.37%)	27 (22.5%)	4 (3.33%)
Depression	59 (49.17%)	43 (35.83%)	16 (13.3%)	2 (1.7%)
Anxiety	64 (53.33%)	45 (37,47%)	10 (8.4%)	1 (0.8%)

**Table 4:** Treatment-mediated long COVID neuropsychiatric symptom severity in people undergoing health resort treatment.

**Concentration Disorders (points)**	**Pre-Treatment Measurement**			**Post-Treatment Measurement**			***p* Value ^1^**
	**Min**	**Max**	**Mean ± SD**	**Min**	**Max**	**Mean ± SD**	
Whole group (*n* = 120)	0	8	2.47 ± 2.47	0	5	0.86 ± 1.22	*p* < 0.001
Women (*n* = 69)	0	8	2.93 ± 2.53	0	5	1.03 ± 1.35	*p* < 0.001
Men (*n* = 51)	0	8	1.84 ± 2.26	0	3	0.63 ± 0.98	*p* < 0.001
*p* value ^2^	*p* > 0.05			*p* > 0.05			
**Memory Disorders (Points)**	**Pre-Treatment Measurement**			**Post-Treatment Measurement**			***p* Value ^1^**
	**Min**	**Max**	**Mean ± SD**	**Min**	**Max**	**Mean ± SD**	
Whole group (*n* = 120)	0	8	2.68 ± 2.5	0	5	1 ± 1.4	*p* < 0.001
Women (*n* = 69)	0	8	3.20 ± 2.48	0	5	1.15 ± 1.42	*p* < 0.001
Men (*n* = 51)	0	8	1.96 ± 2.36	0	5	0.8 ± 1.36	*p* < 0.001
*p* value ^2^	*p* < 0.001			*p* > 0.05			
**Headache (Points)**	**Pre-Treatment Measurement**			**Post-Treatment Measurement**			***p* Value ^1^**
	**Min**	**Max**	**Mean ± SD**	**Min**	**Max**	**Mean ± SD**	
Whole group (*n* = 120)	0	8	1.48 ± 2.04	0	5	0.42 ± 0.89	*p* < 0.001
Women (*n* = 69)	0	8	1.88 ± 2.16	0	5	0.57 ± 1.06	*p* < 0.001
Men (*n* = 51)	0	7	0.94 ± 1.75	0	3	0.22 ± 0.54	*p* < 0.01
*p* value ^2^	*p* < 0.05			*p* > 0.05			
**Dizziness (Points)**	**Pre-Treatment Measurement**			**Post-Treatment Measurement**			***p* Value ^1^**
	**Min**	**Max**	**Mean ± SD**	**Min**	**Max**	**Mean ± SD**	
Whole group (*n* = 120)	0	7	1.39 ± 1.94	0	5	0.34 ± 0.76	*p* < 0.001
Women (*n* = 69)	0	7	1.84 ± 2.17	0	5	0.39 ± 0.81	*p* < 0.001
Men (*n* = 51)	0	5	0.78 ± 1.36	0	3	0.27 ± 0.7	*p* < 0.001
*p* value ^2^	*p* < 0.05			*p* > 0.05			
**Sleep Disorders (Points)**	**Pre-Treatment Measurement**			**Post-Treatment Measurement**			***p* Value ^1^**
	**Min**	**Max**	**Mean ± SD**	**Min**	**Max**	**Mean ± SD**	
Whole group (*n* = 120)	0	8	2.47 ± 2.23	0	5	0.86 ± 1.25	*p* < 0.001
Women (*n* = 69)	0	8	2.77 ± 2.25	0	5	0.86 ± 1.26	*p* < 0.001
Men (*n* = 51)	0	7	2.18 ± 2.15	0	5	0.86 ± 1.25	*p* < 0.001
*p* value ^2^	*p* > 0.05			*p* > 0.05			
**Paresthesia (Points)**	**Pre-Treatment Measurement**			**Post-Treatment Measurement**			***p* Value ^1^**
	**Min**	**Max**	**Mean ± SD**	**Min**	**Max**	**Mean ± SD**	
Whole group (*n* = 120)	0	10	2.47 ± 2.23	0	4	0.63 ± 0.89	*p* < 0.001
Women (*n* = 69)	0	10	2.78 ± 2.32	0	4	0.75 ± 0.96	*p* < 0.001
Men (*n* = 51)	0	8	2.04 ± 2.04	0	3	0.47 ± 0.76	*p* < 0.001
*p* value ^2^	*p* > 0.05			*p* > 0.05			
**Depression (Points)**	**Pre-Treatment Measurement**			**Post-Treatment Measurement**			***p* Value ^1^**
	**Min**	**Max**	**Mean ± SD**	**Min**	**Max**	**Mean ± SD**	
Whole group (*n* = 120)	0	9	1.5 ± 1.92	0	9	0.47 ± 1.09	*p* < 0.001
Women (*n* = 69)	0	8	1.78 ± 1.92	0	3	0.52 ± 0.88	*p* < 0.001
Men (*n* = 51)	0	9	1.12 ± 1.87	0	9	0.39 ± 1.34	*p* < 0.001
*p* value ^2^	*p* < 0.05			*p* > 0.05			
**Anxiety (Points)**	**Pre-Treatment Measurement**			**Post-Treatment Measurement**			***p* Value ^1^**
	**Min**	**Max**	**Mean ± SD**	**Min**	**Max**	**Mean ± SD**	
Whole group (*n* = 120)	0	8	1.17 ± 1.62	0	3	0.33 ± 0.68	*p* < 0.001
Women (*n* = 69)	0	8	1.46 ± 1.81	0	3	0.48 ± 0.82	*p* < 0.001
Men (*n* = 51)	0	4	0.76 ± 1.21	0	1	0.14 ± 0.35	*p* < 0.001
*p* value ^2^	*p* > 0.05			*p* > 0.05			

**Legend**: *p* value ^1^—intragroup comparison; *p* value ^2^—intergroup comparison.

**Table 5:** Comparison of the magnitude of changes mediated by the health resort treatment in women and men with long COVID.

∆Symptom	Women (Mean ± SD)	Men (Mean ± SD)	*p* Value
Concentration disorders	1.9 ± 2.03	1.22 ± 1.84	0.06
Memory disorders	2.06 ± 1.97	1.16 ± 1.9	0.006
Headaches	1.32 ± 1.71	0.73 ± 1.56	0.06
Dizziness	1.45 ± 1.91	0.51 ± 0.97	0.02
Sleep disorders	1.91 ± 2.01	1.31 ± 1.71	0.1
Paresthesia	2.03 ± 1.87	1.57 ± 1.69	0.18
Depression	1.26 ± 1.59	0.73 ± 1.18	0.07
Anxiety	0.99 ± 1.49	0.63 ± 1.06	0.29

**Table 6:** Characteristics of patients in groups I and II.

	Group I (*n* = 70)			Group II (*n* = 50)			
		*n*	%		*n*	%	
	Women	43	61.43	Women	26	52%	
	Men	27	38.57	Men	24	48%	
	**Min**	**Max**	**Mean ± SD**	**Min**	**Max**	**Mean ± SD**	***p* value**
Age (years)	42	77	63.73 ± 8.03	42	79	64.88 ± 9.53	*p* > 0.05
Body weight (kg)	55	120	85.11 ± 15.7	57	125	87.02 ± 14.74	*p* > 0.05
Body height (m)	1.5	1.95	1.67 ± 0.09	1.51	1.85	1.67 ± 0.08	*p* > 0.05
BMI (kg/m^2^)	21.45	46.66	30.37 ± 5.05	21.97	46.47	31.19 ± 5.16	*p* > 0.05
Systolic blood pressure (mmHg)	105	165	138.11 ± 13.22	110	160	141.74 ± 13.36	*p* > 0.05
Diastolic blood pressure (mmHg)	55	95	78.93 ± 8.11	63	95	81.68 ± 6.41	*p* > 0.05
Treatment duration (day)	17	47	25.19 ± 6.83	20	41	23.4 ± 5.2	*p* < 0.05

**Table 7:** Treatment-mediated long COVID neuropsychiatric symptom severity depending on application of balneological factors.

**Concentration Disorders (Points)**	**Pre-Treatment Measurement**			**Post-Treatment Measurement**			***p* Value ^1^**
	**Min**	**Max**	**Mean ± SD**	**Min**	**Max**	**Mean ± SD**	
Group I (*n* = 70)	0	8	2.93 ± 2.49	0	5	0.99 ± 1.3	*p* < 0.001
Group II (*n* = 50)	0	7	1.82 ± 2.3	0	4	0.68 ± 1.08	*p* < 0.001
*p* value ^2^	*p* < 0.05			*p* > 0.05			
**Memory Disorders (Points)**	**Pre-Treatment Measurement**			**Post-Treatment Measurement**			***p* Value ^1^**
	**Min**	**Max**	**Mean ± SD**	**Min**	**Max**	**Mean ± SD**	
Group I (*n* = 70)	0	8	2.97 ± 2.54	0	5	1.00 ± 1.40	*p* < 0.001
Group II (*n* = 50)	0	7	2.26 ± 2.41	0	5	1.00 ± 1.4	*p* < 0.001
*p* value ^2^	*p* > 0.05			*p* > 0.05			
**Headache (Points)**	**Pre-Treatment Measurement**			**Post-Treatment Measurement**			***p* Value ^1^**
	**Min**	**Max**	**Mean ± SD**	**Min**	**Max**	**Mean ± SD**	
Group I (*n* = 70)	0	8	1.67 ± 2.14	0	5	0.49 ± 1.02	*p* < 0.001
Group II (*n* = 50)	0	7	1.22 ± 1.88	0	3	0.32 ± 0.68	*p* < 0.001
*p* value ^2^	*p* > 0.05			*p* > 0.05			
**Dizziness (Points)**	**Pre-Treatment Measurement**			**Post-Treatment Measurement**			***p* Value ^1^**
	**Min**	**Max**	**Mean ± SD**	**Min**	**Max**	**Mean ± SD**	
Group I (*n* = 70)	0	7	1.56 ± 2.00	0	5	0.36 ± 0.82	*p* < 0.001
Group II (*n* = 50)	0	7	1.16 ± 1.84	0	3	0.32 ± 0.68	*p* < 0.001
*p* value ^2^	*p* > 0.05			*p* > 0.05			
**Sleep Disorders (Points)**	**Pre-Treatment Measurement**			**Post-Treatment Measurement**			***p* Value ^1^**
	**Min**	**Max**	**Mean ± SD**	**Min**	**Max**	**Mean ± SD**	
Group I (*n* = 70)	0	8	2.51 ± 2.13	0	4	0.79 ± 1.12	*p* < 0.001
Group II (*n* = 50)	0	8	2.52 ± 2.36	0	5	0.96 ± 1.41	*p* < 0.001
*p* value ^2^	*p* > 0.05			*p* > 0.05			
**Paresthesia (Points)**	**Pre-Treatment Measurement**			**Post-Treatment Measurement**			***p* Value ^1^**
	**Min**	**Max**	**Mean ± SD**	**Min**	**Max**	**Mean ± SD**	
Group I (*n* = 70)	0	10	3.01 ± 2.09	0	4	0.77 ± 0.97	*p* < 0.001
Group II (*n* = 50)	0	9	1.7 ± 2.21	0	3	0.44 ± 0.73	*p* < 0.001
*p* value ^2^	*p* < 0.001			*p* > 0.05			
**Depression (Points)**	**Pre-Treatment Measurement**			**Post-Treatment Measurement**			***p* Value ^1^**
	**Min**	**Max**	**Mean ± SD**	**Min**	**Max**	**Mean ± SD**	
Group I (*n* = 70)	0	7	1.69 ± 1.73	0	3	0.49 ± 0.85	*p* < 0.001
Group II (*n* = 50)	0	9	1.24 ± 2.15	0	9	0.44 ± 1.37	*p* < 0.001
*p* value ^2^	*p* < 0.05			*p* > 0.05			
**Anxiety (Points)**	**Pre-Treatment Measurement**			**Post-Treatment Measurement**			***p* Value ^1^**
	**Min**	**Max**	**Mean ± SD**	**Min**	**Max**	**Mean ± SD**	
Group I (*n* = 70)	0	7	1.36 ± 1.61	0	3	0.39 ± 0.79	*p* < 0.001
Group II (*n* = 50)	0	8	0.9 ± 1.61	0	2	0.26 ± 0.49	*p* < 0.001
*p* value ^2^	*p* > 0.05			*p* > 0.05			

**Legend**: *p* value ^1^—intragroup comparison; *p* value ^2^—intergroup comparison.

**Table 8:** Comparison of the magnitude of changes mediated by the health resort treatment in groups of patients with dependency on application of balneological factors.

∆Symptom (Points)	Group I (Mean ± SD)	Group II (Mean ± SD)	*p* Value
Concentration disorders	1.94 ± 1.99	1.14 ± 1.86	0.02
Memory disorders	1.97 ± 1.97	1.26 ± 1.95	0.02
Headaches	1.19 ± 1.72	0.9 ± 1.59	0.39
Dizziness	1.2 ± 1.7	0.84 ± 1.56	0.2
Sleep disorders	1.73 ± 1.93	1.56 ± 1.88	0.65
Paresthesia	2.24 ± 1.72	1.26 ± 1.8	0.0004
Depression	1.2 ± 1.36	0.8 ± 1.55	0.04
Anxiety	0.97 ± 1.31	0.64 ± 1.35	0.18

## Data Availability

The data that support the findings of this study are available from the corresponding author.
